# Human Factors Affecting Logging Injury Incidents in Idaho and the Potential for Real-Time Location-Sharing Technology to Improve Safety

**DOI:** 10.3390/safety4040043

**Published:** 2018-10

**Authors:** Soren M. Newman, Robert F. Keefe, Randall H. Brooks, Emily Q. Ahonen, Ann M. Wempe

**Affiliations:** 1 Office of Grant and Project Development, College of Agricultural and Life Sciences, University of Idaho, Moscow, ID 83844, USA; 2 Department of Forest, Rangeland and Fire Sciences, College of Natural Resources, University of Idaho, Moscow, ID 83844, USA; robk@uidaho.edu (R.F.K.); rbrooks@uidaho.edu (R.H.B.); awempe@uidaho.edu (A.M.W.); 3 Richard M. Fairbanks School of Public Health, Indiana University, Indianapolis, IN 46202, USA; eqahonen@iu.edu

**Keywords:** cable logging, forestry workers, logging safety, Global Navigation Satellite System-Radio Frequency (GNSS-RF), location-based services, internet of things, interviews, survey, mixed methods, situational awareness

## Abstract

Human factors, including inadequate situational awareness, can contribute to fatal and near-fatal traumatic injuries in logging, which is among the most dangerous occupations in the United States. Real-time location-sharing technology may help improve situational awareness for loggers. We surveyed and interviewed professional logging contractors in Idaho to (1) characterize current perceptions of in-woods hazards and the human factors that lead to injuries; (2) understand their perspectives on using technology-based location-sharing solutions to improve safety in remote work environments; and (3) identify logging hazard scenarios that could be mitigated using location-sharing technology. We found production pressure, fatigue, and inexperience among the most-common factors contributing to logging injuries from the perspective of participants. Potential limitations of location-sharing technology identified included potential for distraction and cost. Contractors identified several situations where the technology may help improve safety, including (1) alerting workers of potential hand-faller injuries due to lack of movement; (2) helping rigging crews to maintain safe distances from yarded trees and logs during cable logging; and (3) providing a means for equipment operators to see approaching ground workers, especially in low-visibility situations.

## 1. Introduction

Logging consistently ranks among the most dangerous occupations [1,2]. With a rate of 136 fatal injuries per 100,000 workers (91 fatalities total) in 2016, logging workers had by far the highest fatal injury rate in the United States [3]. Human factors, or “environmental, organizational and job factors, and human and individual characteristics which influence behavior at work in a way which can affect health and safety,” are among the variables contributing to hazardous working conditions on logging operations [4]. For example, Slappendel et al. identified factors contributing to logging injuries from the literature published from 1970–1991, which they organized into four categories: (1) personal characteristics (judgement and decision making; skill and technique; experience, education, and training; and age); (2) machinery, tools, and equipment (chain saw vibration and noise, chain sawkick back, forest machine design, protective equipment, and maintenance); (3) work organization (task demands, mechanization, piecework vs. salary payment, contracting/subcontracting, and company size); and (4) physical environment (climate, lighting, terrain, and flora) [5]. Fatigue related to long shifts, reduced sleep, and fast-paced, intensive work can also be a factor in logging injuries [6,7], along with financial pressures and inadequate training [8].

**Figure 1.**
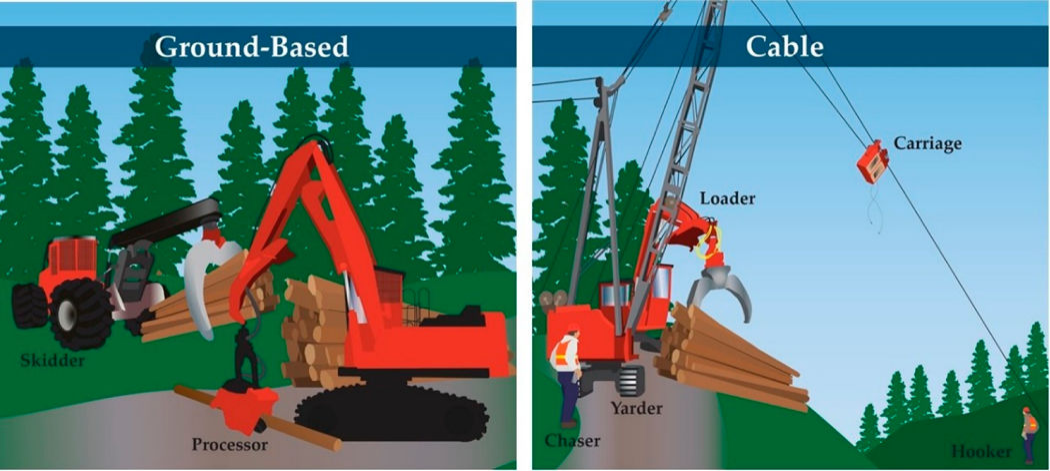
In fully mechanized ground-based logging systems (**left**), most workers are protected in enclosed cabs, which can reduce risk of injury. On cable logging operations in the US Inland Northwest, which occur on slopes greater than 40%, ground workers are intermingled with heavy equipment. Hand-felling of trees using chainsaws is still the primary method of harvesting on cable operations, while mechanized felling is typically used in ground-based systems. The cable system (**right**) figure was adapted from an earlier version [13].

Improvements in basic safety measures in the US logging industry, such as use of personal protective equipment (PPE), have been made in part due to workers’ compensation insurance requirements [9]. Mechanization has helped reduce fatalities and near-fatal injuries by moving more workers into enclosed, protected machine cabs in ground-based logging systems, which operate on moderately sloped topography [10,11]. While the logging industry has experienced a trend of fewer injuries with greater mechanization, the severity of injuries has increased [9]. Emerging mechanized equipment for steep-slope harvesting is growing in use [12] but has not been widely adopted in the US Inland Northwest where exposed ground workers are still common, especially on cable logging operations [13]. Cable logging is regularly used on slopes greater than 40%, which constitute approximately half of logging operations in the region [14]. As cable logging occurs on steep slopes, manual tree felling is most commonly done by workers with chainsaws, and rigging crew workers set chokers (steel cables) around logs by hand before they are yarded up hill to a log landing (Figure 1).

With a mix of ground workers and heavy equipment working alongside one another, often on steep slopes, several potential hazardous scenarios exist. These include injuries resulting from falling trees or trees moved by equipment or cables; kickback or other cutting injuries associated with chainsaws; falling live tree and snag (dead standing tree) hazards; logs, rocks, and other objects that are dislodged and roll down slope; rollover injuries caused when equipment tips over on steep slopes; and pinch-point injuries that occur beneath overhead equipment (e.g., the skyline carriage) or behind rotating machines (e.g., loaders, processors, and swing yarders). Several studies have found claims are most often filed for injuries related to being struck by or against objects, such as tree parts, snags, and logs, with many fatalities related to head injuries caused by falling trees [9,15,16]. Injuries related to falling from equipment while doing maintenance and repair are among the most-common injuries on mechanized logging operations [8,9,11]. ‘Not seen’ injury incidents occur because equipment operators face the difficult dual task of being aware of ground workers’ locations while focused on operating heavy machinery [17].

Situational awareness (SA) can be described as the “state of understanding what is happening in an event with many actors and moving parts” [18] (p. 1079). Endsley posits SA has three levels: (1) perception of elements in the environment (e.g., knowledge of terrain and where workers are); (2) comprehension of the current situation (i.e., the ability integrate and understand the meaning of the individual elements perceived in level 1); and (3) projection of future states, or the ability to use the integrated information from level 2 to make decisions consistent with operation goals [19]. As Endsley explains

Acquiring and maintaining SA becomes increasingly difficult, however, as the complexity and dynamics of the environment increase. In dynamic environments, many decisions are required across a fairly narrow space of time, and tasks are dependent on an ongoing, up-to-date analysis of the environment. Because the state of the environment is constantly changing, often in complex ways, a major portion of the operator’s job becomes that of obtaining and maintaining good SA. [19] (p. 33)

Logging operations often present dynamic environments wherein maintaining SA is difficult, yet critical for worker safety. Location-sharing devices, such as new Global Navigation Satellite System-Radio Frequency (GNSS-RF) technologies that share geographic coordinates and radio-frequency identification (RFID) transmitters capable of local relative positioning of worker proximity to equipment, have potential to increase workers’ SA on logging operations. GNSS-RF devices that facilitate location sharing in off-the-grid areas without cellular service include receivers that synchronize with mobile phones or tablets using Bluetooth and transmit GNSS locations throughout local networks, as well as dedicated radios with GNSS capabilities. These latter devices originated for military applications but are increasingly being considered for worker safety uses in natural resources.

Keefe et al. proposed the development of a system that would allow equipment operators to see locations of ground workers and other equipment on a digital display in real time using location-sharing devices [20]. This system would increase SA during logging, thereby reducing the incidence of fatal and near-fatal injuries. Virtual geofences encompassing high-risk areas on logging operations have been evaluated as a mechanism to detect and alert operators to the presence of ground workers in hazardous areas [13,21]. Zimbelman et al. expanded this concept to include detection of workers and equipment in motion through real-time proximity analysis [22]. The movements of loggers and their proximity to multiple hazards during active logging operations was evaluated on three timber sales in northern Idaho [13]. This study found ground workers spent 23% to 53% of their time in hazardous areas.

When little forest canopy is present, GNSS positions sent at intervals of one location every 2.5 s are sufficiently fast to characterize equipment movements, like the swinging boom of a log loader or processor [23]. However, GNSS system positioning error and the radio signals used to transmit those location data in ad-hoc networks in the woods are affected by forest canopy characteristics and topography [24]. Therefore, GNSS-based geofences should generally not be used to provide fine-resolution safety alerts to operators [13]. Real-time location sharing may still help improve general SA on the jobsite and has been identified as an important area for research and development in forest operations [13,20,25].

Several studies have established the technical basis for the kinds of situations in which it may be appropriate to use real-time location sharing of worker and equipment GNSS coordinates to improve safety on logging operations [13,21,22,24]. Yet, for these systems to be further developed and used in practice, it is necessary to understand professional loggers’ perspectives on potential uses and limitations of location sharing and this has not yet been explored in the literature. Loggers are best qualified to identify tasks and patterns of movement during common logging activities that would most benefit from use of location sharing to improve SA and assist with injury avoidance, detection, and response. Their input can best inform the design of technical field experiments to quantify location-sharing accuracy and suitability for use in the hazard and response scenarios identified, and provide background for subsequent surveys to quantify and evaluate more nuanced hypotheses about technology adoption among this worker group. Identifying and characterizing human factors that currently contribute to logging injury incidents is an important next step for informed development of technology-based interventions intended to improve safety.

Therefore, this paper contributes to the literature by using in-depth interviews and surveys with professional logging contractors to (1) characterize current situations, conditions, and human factors that lead to logging injury incidents to identify hazard situations that could be mitigated using GNSS-RF or other location-sharing technologies; and (2) understand loggers’ perspectives on potential uses and limitations to using real-time location-sharing devices to improve safety in remote work environments. In addition to characterizing loggers’ perspectives on safety hazard situations, associated human factors, and the appropriateness of adopting real-time location-sharing technology, we used survey and interview results to identify, develop, and prioritize specific hazard scenarios for subsequent field experiments evaluating use of GNSS-RF technology on active logging operations.

## 2. Methods

This study was approved by the University of Idaho Institutional Review board (project 15–797). We adopted a concurrent mixed-methods approach, meaning we gathered interview and survey data simultaneously in the same period (February to April 2016). The purpose of the mixed-method approach was twofold: (1) Complementarity (e.g., a strength of quantitative survey data is that it can provide breadth while qualitative interview data can provide explanation and depth) and (2) triangulation (i.e., ability to compare quantitative and qualitative findings) [26]. The methodology for each approach is described in depth below.

### 2.1. Interview Methods

We identified interviewees through their participation in the Idaho Logger Education to Advance Professionalism (LEAP) program. Many Idaho loggers participate in LEAP in part because it fulfills Idaho Pro-Logger accreditation requirements. Major forest landowners require Idaho logging contractors to have Pro-Logger accreditation to retain certification under several national and international sustainability programs, such as the Sustainable Forestry Initiative (SFI) and Forest Stewardship Council (FSC).

We purposefully selected 75 potential interviewees from a list of all 677 loggers who participated in Idaho LEAP programs from 2004 to 2015. The aim of this maximum variation sample was to ensure a range of perspectives and characteristics would be represented. For example, it was important both ground and cable logging system experiences were included since these systems are both used extensively in the study region. Once we had interviewed all willing participants from the purposeful sample, our data included a range of perspectives, but we had not yet reached theoretical saturation, which occurs when “gathering fresh data no longer spark new theoretical insights, nor reveal new categories or concepts” [27] (p. 167). Therefore, we expanded our interview sampling frame by randomly selecting loggers from the full list. Interviewing continued until we reached theoretical saturation.

We conducted in-depth interviews in person with 5 participants and by phone with 36 participants for a total of 41 interviews. Interviews lasted approximately 30–60 min. All interviews were audio recorded and transcribed except for one interviewee who preferred not to be recorded. In that case, detailed notes were used in the analysis instead of a transcript.

Interviews were semi-structured with open-ended questions and covered four primary topics: hazardous situations and conditions as well as common injuries; human factors that contribute to injury incidents; potential suitable and unsuitable applications of real-time location-sharing technologies for logging operations; and interest, concerns, and potential barriers to adopting these technologieson logging operations. Before the series of questions related to location-sharing technology, the interviewer described the technology and answered the interviewee’s questions. Some participants said they had seen demonstrations of real-time location-sharing technology at LEAP trainings. A brief first set of questions helped us track participant characteristics (years of logging experience, type of logging systems worked in, land ownership type worked in, and company size).

Semi-structured interviews allow flexibility to explore participants’ specific knowledge, experiences, and perspectives related to pre-defined topics. We asked participants the same questions, but not necessarily in the same order, and follow-up questions were developed spontaneously. The corresponding author conducted 28 interviews, and two student research assistants conducted the other 13 after participating in training to ensure quality and consistency across interviewers. The corresponding author continued training and supporting the student assistants throughout the data collection process, including meeting regularly to debrief and to discuss and compare preliminary analytical observations.

The corresponding author conducted the formal analysis of interview data using ATLAS.ti qualitative data analysis software. The analysis involved systematically identifying, labeling, and organizing themes in the data following an inductive approach [28]. Initial coding was the first phase of the analysis, which occurred simultaneously with data collection. Initial coding involves developing codes, or short and precise labels, that categorize and summarize segments of data [27]. Once all data was collected and initial coding completed, the next step was to compare, refine, and sort initial codes into themes. Next, the corresponding author conducted focused coding wherein the refined codes capturing main themes were used to sort through the interview data. The final step was to identify quotations that illustrated the most salient themes.

### 2.2. Survey Methods

We collected survey data using convenience sampling at six LEAP Update workshops held throughout northern Idaho in spring 2016 (Table 1). The self-administered survey used closed-and open-ended questions to collect information on personal protective equipment (PPE) use, perspectives on potential factors contributing to logging injuries and common hazardous situations, and demographic and employment information. We identified the factors contributing to injuries and common hazardous situations covered on the survey based on literature reviews and Fatality Assessment and Control Evaluation (FACE) program reports [29]. Respondents were asked “Based on your experience, on a scale from 1 to 5, how common is it for the following factors to contribute to injuries on logging operations in the Inland Northwest?” and “Based on your experience, on a scale from 1 to 5, how common are the following hazardous situations on logging operations in the Inland Northwest?” Respondents answered these questions on a five-point scale ranging from 1 (“not at all common”) to 5 (“very common”) and had the opportunity to list and describe additional hazardous situations and factors in a follow-up open-ended question. One purpose of these questions was to identify and prioritize safety situations for the field experiment component of the broader project. We calculated descriptive statistics for the survey data and created tables using Excel.

While the interview and survey methods were designed to be complementary, because the interviews were semi-structured with open-ended questions and conducted concurrently with the survey, the themes that emerged from the interview data were broader than the closed-ended answer options presented to respondents on the survey and some survey answer options did not emerge as salient interview themes. We now present the results.

## 3. Results

### 3.1. Participant Characteristics

#### 3.1.1. Interviewee Characteristics

Interviewees had an average of 32 years of logging experience, ranging from 7 to 61 years (Table 2). While most had experience with multiple types of logging systems—either through past work experiences or because they currently owned or worked for companies with capacity for multiple types—the majority (35) said they currently do ground-based, mechanical logging and 9 said they use a cable system (instead of or in addition to a ground-based system). When asked where they primarily work, 38 interviewees’ (93%) responses included northern Idaho, 8 (20%) included eastern Washington, 2 (5%) included western Montana, and 1 (2%) participant said his company works throughout Washington, Oregon, Idaho, and Montana.

Many interviewees had past or current experiences working on a variety of forest land ownership types, but the greatest number had experience logging on industrial (27) and non-industrial private land. Interviewees tended to represent small contractors: 24 respondents said the company they worked for had 5 or fewer employees and another 7 worked for companies with 6 to 10 employees. One interviewee was female, and 40 were male.

#### 3.1.2. Survey Respondent Characteristics

A total of 272 loggers responded to the survey. Table 3 describes respondents’ characteristics. When asked “What is your job title?”, most respondents selected multiple answer options, but the majority (74%) indicated their job involves operating equipment, and 33% identified their duties as including timber falling. The average age of respondents was 48 and they had an average of 28 years of work experience in the logging industry. Just over half (53%) said a high school diploma or GED was the highest level of education they had completed. Most respondents (65%) worked for or owned companies with five or fewer employees, counting themselves (median = 13 employees).

### 3.2. Factors That Contribute to Logging Injury Incidents

#### 3.2.1. Forest and Landscape Conditions

When asked to describe the primary physical environmental factors that lead to hazardous work conditions on logging operations like theirs, interviewees’ most common responses included weather leading to slick conditions (rain, ice, and snow), steep terrain, as well as trees and snags: “The mostdangerous thing that we really work around is dead trees and real steep ground.” Other hazards interviewees described were often related to these factors. For example, many interviewees said it is harder to move quickly on steep ground to get out of the way of objects rolling down slope: “Steep ground is so much harder to get around on [and] then as you drag the logs up the hill … you’ve got rocks, log butts, and everything else rolling down.” Steep, sometimes rocky slopes, interviewees said, contribute to equipment rollovers, rolling objects as well as mobility and visibility impediments—for example, on cable operations the yarder operator positioned at the top of the slope may not be able to see the ground workers hooking the logs to the cable downslope. A few interviewees mentioned limited visibility due to dust, fog, or smoke.

The survey included only one factor related to forest and landscape conditions: The survey asked, “Based on your experience, on a scale from 1–5 (1 = not at all common, 5 = very common), how common is it for reduced awareness due to weather conditions or smoke to contribute to injuries on logging operations in the Inland Northwest?” The average response was 2.47 (SD = 1.08, *n* = 266).

#### 3.2.2. Structural Drivers of Working Conditions

When describing factors that contribute to injury incidents on logging operations, nearly all interviewees emphasized the importance of knowing what is going on around them (i.e., SA): “Everybody’s got to be aware and everybody in the machine needs to be … cognizant of what’s around them all the time—where the guys are.” Although researchers did not ask interviewees directly about situational awareness in the part of the interview that elicited this theme and interviewees did not use the term “situational awareness” specifically, as the illustrative quotes presented convey, interviewees were describing what the researchers conceptualized as situational awareness. Some felt it is impossible for workers to have the level of SA necessary to avoid all injuries:

A lot of [injuries happen] from not being terribly observant, but you can’t always be observant enough if you’ve got to be out here producing enough to make a living. We’re not being paid enough to buzz around there with a spyglass to figure out what you might have above your head all the time.

Most interviewees attributed reduced SA to production pressure related to high capital expenses and the need to complete harvests within the contract price: “They want to get as much done per day as they can, and so they start cutting corners instead of paying attention to what’s going on.” Table 4 presents factors related to structural conditions that contribute to injuries on logging operations in the Inland Northwest listed from most to least common according to survey results. “Pressure to harvest as much as possible as quickly as possible” was not only the factor with the highest mean for those related to structural conditions specifically, but for all factors that contribute to injuries we asked about on the survey. These findings support prior research in operational forestry indicating production pressure as a key obstacle to improved safety practices [30].

Many interviewees also attributed injury incidents to workers’ level of knowledge and experience: “Nowadays it’s so hard to get a good crew of people that are experienced.” Many said safe logging requires knowledge only developed through experience:

It’s tough to break in people because … they have to be vigilant about what’s going on around them… . The machines, like the yarders, swing and … you’ve got to allow yourself distance. And then again you can’t run down the road a quarter of a mile because … in logging, production is how you make your money. It’s just experience is what it takes.

Some interviewees believed younger workers are less likely to have had experiences that prepare them for logging than previous generations: “You’re getting guys that weren’t around the woods all their lives.” In corresponding survey results, “inadequate job skill level or experience” had an average rating of 2.50 (SD = 1.32, *n* = 264) (Table 4). Relatedly, interview results help contextualize the survey factor “inadequate fitness level” (mean = 2.45, SD = 1.15, *n* = 265). Several interviewees described the aging logging workforce as a factor related to both fitness level and injury incidents: “For us, it’s mainly sprains and leg injuries … we’re just all getting up there in age.”

Fatigue related to working long hours, long commutes, and few days off was another prominent interview theme: “Fatigue becomes a real concern for us in January. The guys are starting to burn out, get tired, cut corners, not get the rest you need.” This result was also supported by survey results where factors such as “inadequate sleep due to long travel time to job site” (mean = 2.75, SD = 1.20, *n* = 265), “physical fatigue/tiredness due to manual labor” (mean = 2.65, SD = 1.16, *n* = 267), and “inadequate sleep due to long shifts” (mean = 2.53, SD = 1.20, *n* = 266) were among the factors contributing to logging injuries with the highest means for the survey overall (Table 4).

The two factors included on the survey related to structural conditions with the lowest means (i.e., “inadequate sleep due to working at night” (mean = 1.99, SD = 1.16, *n* = 260) and “inadequate safety training or safety meetings” (mean = 1.94, SD = 1.08, *n* = 266)) did not emerge as salient interview themes.

#### 3.2.3. Social and Behavioral Factors

In addition to production pressure, several interviewees said occupational culture can contribute to a fast-paced work site: “The culture [was] that you go to the bar at night and you brag about how many logs you got out that day.” Several perceived drug or alcohol use (generally during personal time) as another behavioral factor that contributes to injuries (“The two biggest [safety-related] problems wasn’t out in the woods—it was family problems or sitting in the bar too long during the middle of the week”), although others said drug and alcohol use do not commonly contribute to unsafe work conditions. The latter assertion is more consistent with survey findings reported in Table 5 where “reduced alertness due to alcohol or other substance use” had a relatively low mean of 1.85 (SD = 1.13, *n* = 260). “Distraction due to issues in family or personal life” had the highest mean of the social and behavior-related survey factors (mean = 2.24, SD = 1.10, *n* = 267).

Compared to the structural factors presented in Table 4, the social and behavioral factors we included on the survey generally had lower means (Table 5). “Distraction due to disagreement with co-workers or supervisor” and “distraction due to hunger or dietary needs” were among the factors contributing to injury incidents with the lowest means on the survey overall and were not salient interview themes.

#### 3.2.4. Conditions and Injuries for Ground Workers

There was general agreement among interviewees that ground workers—such as sawyers, hookers, and choker setters—are those most at risk for injuries. The most common types of ground worker injuries interviewees described included those related to working with chainsaws, falling trees, and minor slips, trips and falls: “Probably the most common [injuries] would be … a toss-up between saw cuts and [tree] falling—the hazards of a tree kicking back off the stump, or maybe hitting into another tree and a part of it coming back on the cutter.” In the survey results, “worker slips, trips, or falls” was the factor with the highest mean in terms of injury incidents commonly experienced on logging operations in the Inland Northwest (mean = 2.89, SD = 1.08, *n* = 261) (Table 6). In support of interview results also, injury incidents related to tree falling were among the situations with the highest means from the survey (e.g., “manual faller hit/pinned by a tree they felled” (mean = 2.22, SD = 1.16, *n* = 260), “manual faller cut by chainsaw” (mean = 2.20, SD = 1.14, *n* = 261), and “manual faller hit/pinned by snag or live tree domino effect” (mean = 2.14, SD = 1.16, *n* = 259)).

While the injury incident “ground worker pinned under rolling log dislodged from log deck” had a relatively low mean (mean = 1.72, SD = 0.86, *n* = 255) as far as how common survey respondents viewed this type of incident (Table 6), in related interview results, many described work on the landing near a log deck and in proximity to manual fallers or equipment as the primary hazardous situations for ground workers:

The [machine operator] starts at the back of a road and… high decks [the logs]. If [the logs] roll, they can roll over the top of the guy that got to run up those logs and unhook them. Several … young guys got killed here in my lifetime by decks rolling on them. [It’s important to] know where each other’s at because if [the chain saw operator is] bucking at the back of the [log] deck at the landing and the skidder operator comes and doesn’t know you’re there, the machine guy pushes … the deck out on the [chain saw operator].

The survey injury incident with the second-highest average (i.e., “rigging crew member hit by swinging chokers” (mean = 2.33, SD = 1.19, *n* = 254)) along with several other incidents with relatively high averages (e.g., “rigging crew member hit/pinned by loose log, rock, or other rolling object dislodged uphill by yarded log” (mean = 2.10, SD = 1.11, *n* = 253) and “rigging crew member hit/pinned by choked log” (mean = 2.00, SD = 1.09, *n* = 253)) support interview results related to cable logging. Many interviewees viewed cable systems as presenting the greatest hazards for ground workers because they often work near the cable (where logs and debris are moving, or the cable can break) and downslope from the yarder (where debris can roll):

On line skidding … there’s lots … of stuff that could happen. Sticks sometimes get throwed up in the air and they come down and hit somebody. Sometimes the cable breaks and … whether that carriage weighs 500 or 5000 pounds, it’s going to do a lot of damage if it falls and hits somebody.

Many interviewees mentioned that cable systems typically involve more ground workers since they operate in areas too steep for equipment.

#### 3.2.5. Conditions and Injuries for Equipment Operators

Many interviewees asserted logging has become safer with more workers enclosed in equipment and fewer directly exposed to hazards on the ground: “I think logging has become safer because of all the mechanized equipment.” However, some interviewees described hazards of equipment working around other equipment or near fallers: “We skidded where we had hand fallers, and you want to know where that person is at all times [to] make sure he doesn’t fall a tree on you.” This echoes interview and survey results presented above related to hazards ground workers face working near manual tree fallers. Interviewees commonly cited injuries related to entering and exiting the machine and mechanical maintenance as most typical for machine operators (“Everybody’s inside a protective rollover cage in a machine, so … a smashed finger mechanicing [or] hurting our legs and back climbing around on machines is … the worst of it for us”) or to objects like branches and sticks coming in the cab as a hazard, particularly for operators who are not fully enclosed: “It’s the … stuff coming inside the cab because the cab’s not guarded.” Reinforcing interview results, “worker hurt while working on equipment” was the equipment operator-related injury situation with the highest mean from survey results (mean = 2.17, SD = 1.03, *n* = 263) (Table 7).

While survey results presented in Table 7 show respondents generally viewed equipment rollover-type incidents as relatively uncommon, many interviewees said environmental factors, such as steep terrain combined with wet or icy conditions, are particularly hazardous for equipment operators: “As far as on a ground-based logging like we do, I would think your biggest risk is … the steep ground accidents: Rollovers, skidders tipping over.”

### 3.3. Loggers’ Perspectives on Real-Time Location-Sharing Technology Applications

Several themes emerged related to interviewees’ perspectives on potential applications of real-time location-sharing technology on logging operations. Some interviewees thought it could be used, for better or worse, by supervisors to track workers’ location and productivity: “[If] you’re a boss wanting to keep track where your guys are at, it might be helpful that way.” Many believed the technology would be unnecessary for small-scale logging operations (“[As] a small crew, we know where everybody is”), but useful for large-scale operations with many workers (“I think it would help for bigger outfits with a lot more people … so the equipment people could see the fellas on the ground”). Several interviewees thought the location-sharing technology would be most useful for operations with ground workers (“There is time where [fallers] are working close together, and if they knew where each one was that would definitely help. And of course [on cable systems] always making sure everybody’s out of the way when they start to pull the drag up the hill”) in contrast to operations doing primarily ground-based mechanical logging: “I don’t think on the ground-based operation there would be much benefit … to use it because it’s just all guys in machines and we’re all pretty well connected with CB radios, you know?”

As there are typically more ground workers, some thought location-sharing systems would be most useful on cable operations particularly to help the yarder operator confirm the hookers are out of the way before pulling the logs up (“They’d be really handy on a yarder strip where the guy’s up in the yarder and they can see if the guy’s moved back far enough away from the skid line”). However, several themes did emerge related to situations where location sharing would improve safety for machine operators, particularly when equipment is working near other equipment or ground workers:

On the machine side, it wouldn’t be the end of the world if you had a small screen in there and an alert system because sometimes you need to talk to those buncher [and processor] operators and it’s dark out or it’s snowy, foggy or they’re working in a spot where they do not believe there’s anybody around them. You’re trying to get their attention but it’s too dangerous to get very close. You’ve got to do a weird sneak up on them kind of thing, but the fear that they’re going to whip around and do something crazy is always there because they don’t know you’re there.

**Figure 2.**
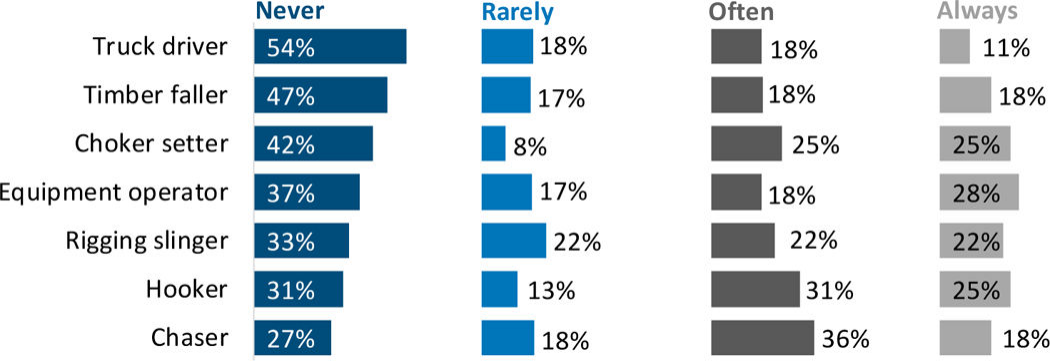
Percent of survey respondents reporting frequency of carrying a two-way radio, by job title.

With the exception of manual fallers who many said could check a device just before they fall a tree to ensure everyone is out of the way, interviewees generally thought there was more value to equipment operators and supervisors being able to monitor ground worker locations than in giving ground workers the ability to monitor others. Many interviewees believed it would be best if ground workers in busy areas were not looking at a device: “I think it would be useful for someone to have GPS on the ground workers, but not the ground workers being able to view what’s on the GPS. I think they need to be focused.” Other prominent interview themes related to potential uses of location-sharing systems on logging operations included staying informed about people working alone and finding injured workers more quickly. This potential application is also supported by survey results related to how often respondents said they carry a two-way radio: a total of 33% of respondents said they “never” carry a two-way radio and 19% said they “rarely” do. Only 26% and 22%, respectively, said they “always” or “often” carry a two-way radio (*n* = 239). Figure 2 shows that many workers, even ground workers without access to a CB radio, reported that they “rarely” or “never” carry a two-way radio. For example, 47% of timber fallers said they “never” carry a two-way radio and another 17% said they “rarely” do. Interview results related to the most-appropriate uses of real-time location sharing are summarized in Table 8.

### 3.4. Barriers to Adopting Real-Time Location-Sharing Technology

The salient interview themes related to potential barriers of logging contractor adoption of real-time location-sharing technology included belief it could be a distraction thereby reducing SA, expense, an invasion of privacy, having limited accuracy, and being difficult to learn. The full list of barrier-related themes with illustrative quotations are presented in Table 9.

## 4. Discussion

### 4.1. Human Factors That Contribute to Logging Injuries

There was high consistency between human factors contributing to logging injuries with the highest means in survey results, and the most salient interview themes, including production pressure, tiredness and fatigue, and lack of work experience.

Considering lack of work experience, it is important to note the left-skewed age demographic of the professional logger population in Idaho. Seventy percent (70%) of those surveyed had more than 21 years of logging experience and 72% were older than 40. The aging workforce and lack of trained younger workers has long been identified as an industry concern [31]. This has implications for safety that are twofold. First, this new cohort replacing retiring, seasoned workers will be less experienced and therefore likely more prone to injuries [32,33]. Second, a positive aspect of a younger cohort of workers moving into the logging industry is the potential for increased use of technology-based solutions to increase safety as younger individuals may be more familiar and accepting of technology [34]. Although we did not specifically address the relationship between age and willingness to adopt this technology, that hypothesis should be evaluated more formally in future research.

It is also important to note that most crews in the region are small: A total of 76% of survey respondents work on crews of 10 individuals or less. This is relevant because, although many of our participants believed real-time location sharing would be most useful to improve SA on large operations, smaller logging crews are associated with increased injury frequency [35].

Our results related to production pressure contribute to a growing literature linking employment conditions to worker health and safety [36,37]. Where occupational safety research has more commonly focused on work conditions (the circumstances resulting “from the nature of the work, including the way work is organized, where and how it is performed, and the physical and social environmental conditions”), there is increasing exploration of employment conditions, which in contrast “describe the nature of the connection between a buyer of labor … and seller of labor” [38]. Our results suggest that the current employment conditions implemented in the study region affect working conditions. This implication is consistent with Lilley et al. who found increased fatigue due to longer working hours, reduced sleep, and a fast-paced environment is a significant injury risk factor in logging in New Zealand [7]. They attributed increased fatigue to structural changes in the industry, such as the introduction of competitive independent contracting and contract payment rates that are tied toproduction rates. Studying this topic in more depth in the context of the Inland Northwest is beyond the scope or focus of the current study and may not be improved directly using location-sharing solutions alone, but may be worth pursuing in longer-term worker safety and health promotion efforts. For example, integration of wearable devices to monitor sleep and other characteristics of logging worker health associated with employment conditions in future research could help to quantify these conditions further.

### 4.2. Concerns about Location-Sharing Technology

The strongest concerns contractors had about use of location-sharing technology included potential distraction from work focus, cost, having to carry additional equipment, privacy concerns, lack of need for the technology among smaller crews and ground-based mechanized operations (contrasted to cable logging operations), and user-friendliness or the steep learning curve. Research showing mobile devices can be a significant distraction to operators of road motor vehicles supports the contention that location-sharing technologies could compromise safety rather than improve it [39]. Therefore, the user interface design for logging applications is an important consideration for reducing distraction and improving device safety. Also, considering participants’ concerns about using location sharing to evaluate productivity, crews adopting this technology will need to integrate it in a way that does not unintentionally exacerbate the production pressure and fatigue many viewed as contributing to injury incidents.

The age demographic of Idaho loggers is also relevant to findings regarding their perspectives on potential barriers to use. Future research should examine whether older forestry workers are less likely to be familiar with smart phones and similar mobile-based solutions for location sharing and, therefore, less willing to adopt technology-based safety practices. The user friendliness of different devices may also affect willingness to use technology-based safety solutions and should be quantified further before developing recommendations about uses of particular devices or software solutions.

### 4.3. Support for Real-Time Location-Sharing Technology

Contractors saw value in the use of location sharing to improve safety for several common situations. Situations that were consistently identified as high priorities included (1) alerting workers to potential hand-faller injuries due to lack of movement; (2) use of location sharing by rigging crews to maintain safe distances from yarded logs during cable logging; and (3) providing a means for equipment operators to see approaching ground workers, especially in low-visibility situations. Location sharing may also be able to increase emergency response and associated communications, as 52% of survey respondents reported they rarely or never carry a two-way radio.

Although not directly identified as a possible use of location-sharing technologies, contractors saw steep slopes as presenting hazardous conditions for ground-based logging. It is important to note that location sharing and related technologies may also help to provide injury avoidance in these situations where rollover injuries are common, much as autonomous vehicles with crash-avoidance systems can identify and respond to oncoming vehicles, road conditions, or other impending hazards. Thus, in future work, it may be important to expand potential interventions associated with location and data sharing to the realm of equipment and automation engineering that considers, for example, vehicular accident avoidance that incorporates interactions between multiple pieces of equipment, and equipment-terrain interactions.

## 5. Conclusions

We have identified several human factors that loggers perceive to be associated with injuries on logging operations in the Inland Northwest. Loggers involved in this study were generally supportive of the use of location-sharing technology to improve SA in specific scenarios. Development of new location-sharing technology and associated safety recommendations will have the highest likelihood of success if they account for the perspectives and concerns of logging contractors. Concerns about technology causing distraction or becoming a burden and cost of implementation are important considerations, and additional survey data collection to inform development of safety recommendations should focus on better characterizing the relative importance of these factors, related tradeoffs, relationships between the logger age demographic and technology adoption, and the potential impact of these factors on behaviors.

Emerging location-sharing devices range in quality, available features, and cost, with the price of individual units ranging from US $100 or less for smartphone-based solutions to US $10,000 or more for advanced, military-grade GNSS-RF radios. Determining the willingness of contractors to purchase devices for safety purposes and feasible price points is another important consideration for future research.

The results of the current study suggest subsequent field experimentation to characterize the suitability of emerging location-sharing technologies for mission-critical safety applications should prioritize improving SA related to the location and safety status of hand fallers and rigging crew workers on cable logging operations. For these individuals who are not protected by equipment cabs, fatal or near-fatal traumatic injuries can occur quickly. When visibility obstructions are present, injury of workers may initially go unnoticed by others at the job site. For that reason, it is important that technology solutions have relatively fast transmission rates (e.g., <1 location per 5 s) to improve the speed of injury detection, interactive hazard notifications, and response times.

## Figures and Tables

**Table 1.** Number of survey respondents, by Idaho Logger Education to Advance Professionalism (LEAP) workshop site and date.

Workshop Site	Date	Number of Respondents
Coeur d’Alene, Idaho	8–9 March 2016	49
Ponderay, Idaho	15–16 March 2016	68
Orofino, Idaho	22–23 March 2016	45
St. Maries, Idaho	29–30 March 2016	37
Moscow, Idaho	4–5 April 2016	49
Moscow, Idaho	28–29 April 2016	24

TOTAL		272

**Table 2.** Interview participant characteristics (*n* = 41).

Characteristic*	%	*n*
Years of logging experience		
Less than 10 years	2%	1
10 to 19 years	10%	4
20 to 39 years	51%	21
40 to 60 years	29%	12
Logging system/work type ^†^		
Ground-based, mechanical logging	85%	35
Cable logging	22%	9
Loading and trucking	7%	3
Helicopter logging	2%	1
Horse logging	2%	1
Site preparation and rehabilitation	2%	1
Forest land ownership work location type ^†^		
Industrial private land	66%	27
Non-industrial private land	61%	25
Federal land	39%	16
State land	37%	15
Number of employees in company		
5 or fewer employees	59%	24
6 to 10 employees	17%	7
11 to 20 employees	10%	4
21 to 50 employees	10%	4
More than 50 employees	2%	1

* All questions were open ended.

† Some interviewees fit into multiple categories.

**Table 3.** Survey respondent characteristics.

Characteristic	%	*n*
Job title * (*n* = 272)		
Equipment operator	74%	202
Timber faller	33%	89
Truck driver	13%	36
Choker setter	12%	33
Logging company owner ^†^	10%	26
Hooker	9%	24
Chaser	7%	19
Rigging slinger	6%	17
Other	8%	22
Age (*n* = 260)		
Younger than 20 years old	0.4%	1
20 to 30 years old	10%	25
31 to 40 years old	19%	48
41 to 50 years old	20%	53
51 to 60 years old	34%	89
61 to 70 years old	14%	36
Older than 70 years old	3%	8
Years of logging experience (*n* = 262)		
5 years or less	6%	17
6 to 10 years	7%	19
11 to 20 years	16%	42
21 to 30 years	31%	81
31 to 50 years	37%	98
More than 50 years	2%	5
Highest degree or level of education (*n* = 255)		
Some high school (no degree) or less	9%	23
High school diploma or GED	53%	136
Some college (no degree)	22%	57
Technical or Associate’s degree	7%	18
Bachelor’s degree	8%	20
Graduate or professional degree	0.4%	1
Number of employees in company (*n* = 261)		
5 or fewer workers	65%	169
6 to 10 workers	11%	30
11 to 20 workers	14%	36
21 to 50 workers	10%	26

* Respondents could select multiple answers.

† “Logging company owner” was not one of the predetermined answer options, but 26 respondents wrote this answer in.

**Table 4.** Survey respondents’ evaluation of how common it is for select factors related to structural conditions to contribute to injury incidents on logging operations in the Inland Northwest, average of 1 to 5 scale (1 = not at all common, 5 = very common).

Factors	Mean	Standard Deviation	*n*
Pressure to harvest as much as possible as quickly as possible	2.98	1.36	263
Inadequate sleep due to long travel time to job site	2.75	1.20	265
Physical fatigue/tiredness due to manual labor	2.65	1.16	267
Inadequate sleep due to long shifts	2.53	1.20	266
Inadequate job skill level or experience	2.50	1.32	264
Inadequate fitness level	2.45	1.15	265
Inadequate sleep due to working at night	1.99	1.16	260
Inadequate safety training or safety meetings	1.94	1.08	266

**Table 5.** Survey respondents’ evaluation of how common it is for select social and behavioral factors to contribute to injury incidents on logging operations in the Inland Northwest, average of 1 to 5 scale (1 = not at all common, 5 = very common).

Factors	Mean	Standard Deviation	*n*
Distraction due to issues in family or personal life	2.24	1.10	267
Distraction due to disagreement with co-workers or supervisor	1.87	0.99	265
Reduced alertness due to alcohol or other substance use	1.85	1.13	260
Distraction due to hunger or dietary needs	1.64	0.88	265

**Table 6.** Survey respondents’ evaluation of how common select injury incidents relevant to ground workers are on logging operations in the Inland Northwest, average of 1 to 5 scale (1 = not at all common, 5 = very common).

Injury Situation	Mean	Standard Deviation	*n*
Worker (any) slips, trips, or falls	2.89	1.08	261
Rigging crew member hit by swinging chokers	2.33	1.19	254
Manual faller hit/pinned by a tree they felled	2.22	1.16	260
Manual faller cut by chainsaw	2.20	1.14	261
Manual faller hit/pinned by snag or live tree domino effect	2.14	1.16	259
Rigging crew member hit/pinned by loose log, rock, or other rolling object dislodged uphill by yarded log	2.10	1.11	253
Rigging crew member hit/pinned by choked log	2.00	1.09	253
Ground worker or operator hit by flying wood or metal projectile due to processing, chipping, or skidding	1.88	0.97	254
Choker setter (hooker) or other worker hit by broken or dislodged skyline, mainline, or choker cable	1.78	0.94	254
Rigging crew member hit by carriage	1.72	0.91	253
Ground worker pinned under rolling log dislodged from log deck	1.72	0.86	255
Manual faller hit/pinned by tree felled by falling partner	1.71	1.03	256
Chaser unhooking chokers hit or pinned by swinging log held by yarder, processor, or loader	1.71	0.86	252
Log rolls off log truck while being loaded and hits worker	1.60	0.86	260
Ground worker pinned between equipment & road cutslope, tree, or rock	1.52	0.74	254

**Table 7.** Survey respondents’ evaluation of how common select injury situations relevant to equipment operators are on logging operations in the Inland Northwest, average of 1 to 5 scale (1 = not at all common, 5 = very common).

Injury Situation	Mean	Standard Deviation	*n*
Worker (any) hurt while working on equipment	2.17	1.03	263
Grapple or cable skidder operator pinned during equipment rollover on hillslope	1.72	0.94	258
Feller-buncher or single-grip harvester operator pinned during equipment rollover on hillslope	1.59	0.85	256

**Table 8.** Summary of interview results related to most appropriate uses of real-time location-sharing technology on logging operations.

Application	Person Monitoring	Description
Large crews	Supervisor	Many interviewees believed large crews have greater need for real-time location sharing than small crews with fewer workers to keep track of.
Supervisor track productivity and safety (bird’s eye view of operation)	Supervisor	Particularly on large crews, many interviewees thought it would be useful for supervisors to see the movement patterns (e.g., amount of time in one position), locations, and productivity of all workers.
Track individuals working alone	Supervisor, others as needed	Useful when any ground worker or equipment operator is working apart from other crew members. For example, no movement could indicate an injury has occurred.
Equipment operators at log landing track ground workers and other equipment at the landing	Equipment operators	Useful for equipment operators to know if ground workers (e.g., chain saw operators) or other equipment are in proximity.
Track ground workers (general)	Supervisor, equipment operators	Many interviewees believed it would be useful for equipment operators and supervisors to see ground worker locations in real time, but in most applications thought it could be a dangerous distraction for ground workers to use the technology to monitor other workers’ positions.
Equipment operators working in proximity to other equipment (general)	Equipment operators	Useful for equipment operators to be alerted if other equipment approaches.
Manual timber fallers check co-workers’ locations before falling a tree	Manual timber faller	Useful for manual tree fallers to check the locations of co-workers to confirm everyone is clear just before falling a tree.
Yarder operator track ground workers working near cable	Yarder operator	Useful for the yarder operator to confirm all ground workers downslope are clear before yarding logs upslope to the landing.
Track truck locations	Supervisor, truck drivers, equipment operators at landing	Useful for supervisors to monitor the location of trucks for safety and productivity. May be useful for truck drivers to track the location of other trucks on forest roads, although some interviewees were concerned drivers would become over reliant on the technology, leading to increased risk taking.

**Table 9.** Salient interview themes related to potential limitations of real-time location-sharing technology.

Theme	Example Quotations
Distraction	“The guy’s watching the screen when he should be watching something else.”
Unnecessary	“I think most guys are pretty well aware where everyone else is at already.”
Cost	“The little guy couldn’t afford to supply that kind of equipment.”
Maintenance	“It’s just a pain in the butt to keep it charged up and ready for use all the time.”
Over reliance	“The guy on the job has got to take responsibility of looking after himself as much as possible.”
Learning	“Just a learning curve and getting used to something new.”
Privacy	“[Loggers] don’t appreciate a tremendous amount of supervision.”
Disuse	“The hard part is getting them to wear it.”
Accuracy	“I’m looking for something that works really good in the canopy of the timber.”
Mandatory use	“I could see…hese safety people…saying, ‘[it’s] mandatory—you’ve got have this.’ And then it just becomes a burden.”
